# Toxin-Antitoxin Systems in Clinical Pathogens

**DOI:** 10.3390/toxins8070227

**Published:** 2016-07-20

**Authors:** Laura Fernández-García, Lucia Blasco, Maria Lopez, German Bou, Rodolfo García-Contreras, Thomas Wood, María Tomas

**Affiliations:** 1 Servicio de Microbiología, Complejo Hospitalario Universitario A Coruña-INIBIC, A Coruña 15006, Spain; laugemis@gmail.com (L.F.-G.); lucia.blasco@gmail.com (L.B.); maria.lopez.diaz@sergas.es (M.L.); german.bou.arevalo@sergas.es (G.B.); 2 Spanish Network for Research in Infectious Diseases (REIPI), Seville 41071, Spain; 3 Departamento de Microbiología y Parasitología, Facultad de Medicina, Universidad Nacional Autónoma de México, Ciudad de México 04510, México; rgarc@bq.unam.mx; 4 Department of Chemical Engineering Pennsylvania State University, University Park, 16802 PA, USA; tuw14@psu.edu; 5 Department of Biochemistry and Molecular Biology, Pennsylvania State University, University Park, 16802 PA, USA

**Keywords:** clinical, pathogens, Toxin-Antitoxin, plasmids, chromosome, resistance, persistance, virulence

## Abstract

Toxin-antitoxin (TA) systems are prevalent in bacteria and archaea. Although not essential for normal cell growth, TA systems are implicated in multiple cellular functions associated with survival under stress conditions. Clinical strains of bacteria are currently causing major human health problems as a result of their multidrug resistance, persistence and strong pathogenicity. Here, we present a review of the TA systems described to date and their biological role in human pathogens belonging to the ESKAPE group (*Enterococcus faecium*, *Staphylococcus aureus*, *Klebsiella pneumoniae*, *Acinetobacter baumannii*, *Pseudomonas aeruginosa* and *Enterobacter* spp.) and others of clinical relevance (*Escherichia coli*, *Burkholderia* spp., *Streptococcus* spp. and *Mycobacterium tuberculosis*). Better understanding of the mechanisms of action of TA systems will enable the development of new lines of treatment for infections caused by the above-mentioned pathogens.

## 1. Introduction

Toxin-antitoxin (TA) systems, which occur in bacteria and archaea, consist of a toxin and an antitoxin, which are respectively stable and unstable components [1,2]. Both components constitute a complex in which the toxin activity or synthesis is inhibited by an antitoxin. Under some conditions, a labile antitoxin is degraded, favoring the action of the toxin by inhibition of essential cellular processes, such as translation, replication, ATP (Adenosine triphosphate) synthesis and cell wall synthesis [2,3,4].

TA systems are either encoded by plasmids or reside in bacterial chromosomes [5]. TA systems in plasmids (TAp) have been associated with plasmid stabilization [6]. It has been theorized that toxin/antitoxin loci serve only to maintain plasmid DNA at the expense of the host organism [7]. Other authors propose that these systems have evolved to favor the competitive ability of plasmids in cell progeny [8]. This hypothesis has been corroborated by computer modeling [8]. However, the role of TA systems encoded by bacterial chromosomes (TAc) is much less well known [9]. Similarly to TAp, the TAc systems have been suggested to play a role in the stabilization of various genetic (pathogenicity islands and prophages) or stress response functions of modular elements of bacterial growth and death [10,11,12].

**Figure 1:**
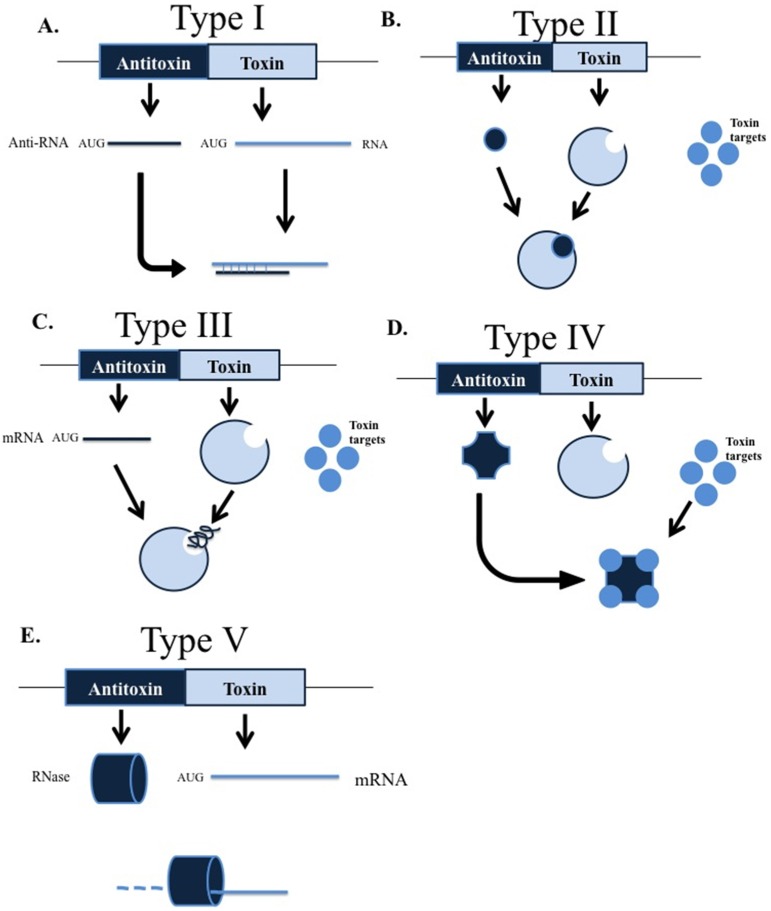
Models representing the interaction between toxins and antitoxins in the different types of toxin-antitoxin (TA) systems. (**A**) Type I: the antitoxin mRNA binding to toxin mRNA, which prevents toxin protein formation; (**B**) type II: a TA complex is formed by the union of toxin and antitoxin proteins; (**C**) type III: a TA complex is formed by the union of toxin protein with antitoxin mRNA; (**D**) type IV: the antitoxin protein binds to the toxin target, blocking its action; (**E**) type V: the antitoxin mRNA encodes an RNAse that degrades the toxin mRNA.

TA systems are currently classified into five groups (types I to V) according to the nature of the antitoxin and the mode of interaction between the toxin and antitoxin [13] (Figure 1). In all cases, the toxins are proteins, while the antitoxins in TA systems types I and III are RNA molecules, and those in TA systems types II, IV and V are proteins. In type I systems, the antitoxin suppresses activity of the toxin protein by binding to its mRNA, whereas in the type II and III TA systems, toxin proteins are blocked by direct binding of antitoxin proteins and the antitoxin RNA, respectively. In type IV TA systems, the antitoxin protein prevents the activity of the toxin by binding to its substrate, and finally, in type V TA systems, the antitoxin RNAse specifically degrades toxin mRNA [13,14].

Hospital-acquired infections are an important problem in the industrialized world, with reported incidence rates of 5% in the United States (U.S.) and 7.1% the European Union (EU) in 2013 [15]. The risk of fatality associated with infections caused by multidrug-resistant (MDR) bacteria (superbugs) is also very high [15]. In recent years, the Infectious Diseases Society of America (IDSA) has highlighted a group of antibiotic-resistant bacteria (*Enterococcus faecium*, *Staphylococcus aureus*, *Klebsiella pneumoniae*, *Acinetobacter baumannii*, *Pseudomonas aeruginosa* and *Enterobacter* spp.), acronymically labelled “the ESKAPE pathogens”, which are capable of “escaping” the biocidal action of antibiotics and mutually representing new paradigms in pathogenesis, transmission and resistance [16]. Several studies have analyzed the function of TAp systems in stabilizing the plasmids that carry resistance genes in clinical pathogens. However, the role of TAc systems in the life of nosocomial bacterial pathogens (ESKAPE) is not well known. TAc systems have been associated with (i) bacterial persistence, by generating slowly-growing cells tolerant to antibiotics and environmental changes, and (ii) biofilm formation, by regulating fimbriae [17]. and by programmed cell death. Nonetheless, there is some controversy regarding the role of TA systems. In a review article, Gerdes et al. related the persistence of *E. coli* as a model organism to type II TA systems. More specifically, these authors suggested that the deletion of type II TA loci significantly reduced the level of persistence [18,19]. However, Kolodkin-Gal et al. studied the involvement of the MazF/MazE and the YafQ/DinJ TA systems in cell death and participation in biofilm formation through novel, as yet unknown mechanism(s) [19].

In this review, we focus on the TA systems that occur in plasmids (TAp) and chromosomes (TAc) of the nosocomial pathogens belonging to the ESKAPE group, as well as other community pathogens that are important in terms of their multi-drug resistance and virulence, i.e., *Escherichia coli*, *Burkholderia* spp., *Streptococcus pneumoniae* and *Mycobacterium tuberculosis*.

The role of toxin-antitoxin systems in clinical pathogens is shown in Table 1.

### 1.1. Pathogens in the ESKAPE Group

#### 1.1.1. *Enterococcus* spp.

*Enterococcus faecalis* and *E. faecium* (order Lactobacillus) are well-known nosocomial pathogens that cause hospital bacteremia, urinary tract infections and surgical wound infections. Enterococci are intrinsically resistant to different antibiotics and can also acquire other types of resistance via mobile genetic elements [78].

Several TAp systems associated with plasmid maintenance have been described in this pathogen. The Par_Ef_ locus of the *E. faecalis* plasmid pAD1 is an RNA-regulated addiction module encoding the Fst peptide toxin. This locus has also been found in other bacteria, such as *Lactobacillus casei* and *S. aureus* [20]. In addition, five genes encoding Fst homologues in *E. faecalis* plasmids have been identified [79]. In 2006, Patel et al. confirmed the role of the Fst toxin in *E. faecalis* strains affecting chromosomal segregation and cell division/peptidoglycan synthesis [21]. The target of this protein is probably located at or near the cell membrane, due to the presence of a hydrophobic stretch of amino acids predicted to form a trans-membrane domain. However, this target either only affects DNA segregation directly or affects both DNA segregation and cell division, and this must be clarified [21]. The omega/epsilon/zeta TAp module has been characterized in plasmid (TAp) and chromosome (TAc) TA systems in pVEF3 plasmids from *E. faecium* resistant to vancomycin (carrying VanA) and persisting on Norwegian poultry farms [23]. The pRUM plasmid encodes multidrug-resistant genes (*van* genes) in *E. faecium.* Bioinformatic analysis of the pRUM sequence enabled identification of a new TAp protein module (Axe/Txe), which is found in multiple bacteria genomes [80]. Co-hybridization studies showed that 90% of the clinical isolates of *E. faecium* were PCR-positive for the pRUM and Axe/Txe TAp systems [22]. The study findings also suggested a genetic relationship between the pRUM replicon and *axe/txe* TAp genes [22]. The axe-txe plasmid system has two promoters, the main one of which has been found upstream of an *antitoxin* gene and is cooperatively regulated by the TA complex. The second was found embedded in the antitoxin CDS and may act in regulating the TA ratio and in modulating toxin transcript stability [81]. The presence of vancomycin resistance has been found to be related to the presence of *axe/txe* TAp genes [22]. Finally, Torill and collaborators [24] investigated the presence of the omega/epsilon/zeta_Ef_ and Axe/Txe TAp systems in relation to the VanA protein, observing that the Axe-Txe TAp system intervenes in maintaining plasmid stability in *E. faecium* and that the omega/epsilon/zeta_Ef_ TAp system stabilizes the plasmid in *Bacillus subtilis*, *E. coli, E. faecium* and *E. faecalis.* These researchers described the high prevalence of TA operons in vancomycin-resistant *Enterococcus* isolates (VRE) relative to sensitive isolates, and their conclusions are consistent with the findings of other authors. The high prevalence of these systems has been related to their role in stable inheritance of the plasmid pool and the relatively large number of VRE plasmids [24].

The Tac systems HigBA_Ef_ and MazEF_Ef_ have been located in the chromosome of *E. faecalis* and *E. faecium* clinical strains and found to be associated with the expression of virulence factors [25].

#### 1.1.2. *Staphylococcus aureus*

*Staphylococcus aureus* is a Gram-positive bacterium (order Bacillales) responsible for an increasing number of nosocomial and community acquired infections. The adaptability of this bacterium to environmental changes, involving temperature, nutritional deprivation or the presence of antibiotics, depends on two component systems: transcription regulatory proteins and regulatory RNAs [29,30].

Until now, only one TAp system has been described in the cCHP91 plasmid in *S. aureus* PeImK_Sa_. This system comprises a toxin, PemK_Sa_, which is a specific ribonuclease for the UAUU sequence, and an antitoxin, PemI_Sa_, which inhibits the toxin by physical interaction. This system is implicated in the maintenance of the plasmid and probably in the regulation of virulence via alteration of the translation of a large pool of genes [26].

**Figure 2:**
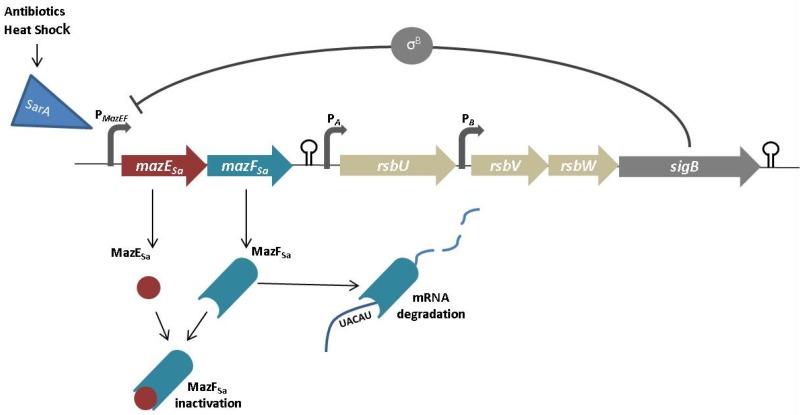
Model of MazEFSa regulation. Transcription of the operon mazES-rsbUVW-sigB is initiated by the mazEF promoter, and transcription of the rsbUVW-sigB genes depends on the activity of the transcriptional terminator downstream of the mazE and mazF genes. This system is negatively regulated by the σB, encoded by sigB, which represses the mazEF promoter. Toxin MazF is an RNAse that degrades the mRNA in the UACAU site. The antitoxin MazE binds and inactivates the toxin MazF. This system is negatively regulated by the σB encoded by sigB.

SrpG1, which belongs to the TxpA-RatA family (type II TA system), is encoded in the mobile genetic element ΦSa3 PI (phage) in *S. aureus* strain N315. The *srpG1* gene overlaps with the SrpF1 non-coding RNA antitoxin. The RNA is constitutively expressed. SrpG1 is negatively regulated by a duplex formation with SrpF1, probably by RNA degradation (Figure 2). Two peptides of different sizes are encoded by SrpG1, yielding a 44-amino acid peptide from the first AUG start codon and, more abundantly, a 31 amino acid peptide from an internal AUG codon. Both peptides are secreted by pore-forming toxins displaying activity against Gram-positive and Gram-negative bacteria, and the longer peptide displays a higher level of lytic activity against human erythrocytes [27,28].

Several TAc systems are located in *S. aureus* strains. In 2012, Sayed and co-workers [29] reported the existence in *S. aureus* of a functional type I TA system previously predicted by computer modelling [82]. This system is located within a pathogenicity island and consists of the SprA1 toxin peptide (PepA1) and SprA1 (AS) RNA antitoxin. PepA1 is induced by oxidative and acidic stress during cell growth and is repressed by an antitoxin type I RNA, the SprA_Sa_ antitoxin. Two explanations for why PepA1 is induced by oxidative and acidic stress have been proposed: one is that under such conditions PepA1 is induced in most of the rapidly dividing internalized bacteria, and slowly dividing bacteria can thus persist and escape the phagolysosomes, the membranes of which will also be damaged by PepA1. Another hypothesis proposes that under oxidative or acidic stress, PepA1 can modulate the activity of membrane proteins involved in iron transport, which together with its hemolytic activity will drive the lysis of the host erythrocytes during infection under iron-limited conditions [29]. The type II TAc systems identified in *S. aureus* are MazEF_Sa_, YefM/YoeB and PemlK_Sa_. MazEF_Sa_ encodes the MazE_Sa_ antitoxin and the MazF_Sa_ RNAse toxin [31,83]. In an in vivo study of *S. aureus*, Fu and coworkers [32]. identified other cleavage sites in *spa*, *sigB* and *hla* mRNAs. It has recently been demonstrated that the *spa* and *rsbW* transcripts are cleaved by the RNAse MazF in the same UACAU recognition sequence. In *S. aureus*, in contrast to the classic TA systems, MazF_Sa_ activity was observed with no changes in the toxin-antitoxin ratio at high levels of toxin, and MazF_Sa_ cleavage was observed in both the presence and absence of MazE. In *S. aureus*, MazEF_Sa_ is located upstream of the *sigB* locus encoding the alternative sigma factor σ^B^, and the adjacent locus encoding another anti-sigma factor *rsbUVW* is implicated in the ability of the bacterium to survive under adverse conditions and in its ability to redirect the RNA polymerase to the transcription of genes involved in the stress response. σ^B^ also plays a role in the expression of virulence genes. This operon, composed by *mazES-rsbUVW-sigB*, is regulated by three promoters. Unlike other TAc systems, MazEF_Sa_ is not self-regulated, as in this case, the P*mazEF* promoter is activated by the SarA regulator and is induced by heat shock or antibiotic stress. In addition, both promoters are upstream *rsbU* and the *rsbV* genes, and two transcriptional terminators are present downstream of the *mazEF_Sa_* genes and the *sigB* gene [28]. This promoter is also negatively regulated by SigB, which acts as a feedback loop for repression of its own transcription [30,32]. Altered sensitivity to β-lactams was observed in a Δ*mazEF*_Sa_ strain, thus suggesting a specific regulatory role for the MazEF_Sa_ locus in β-lactams sensitivity [31]. A second type II TAc system present in *S. aureus* comprises two operons, *yefM/yoeB_Sa1_* and *yefM/yoeB_Sa2_* (previously identified as *axe1-txe1* and *axe2-txe2*), which are homologous to the YefM/YoeB_Ec_ system in *E. coli*. Like YoeB_Ec_, both YoeB_Sa1_ and YoeB_Sa2_ exhibit cellular RNAse activity by inhibiting the initiation of translation and arresting cellular growth [33,34]. Finally, the third system is the omega/epsilon/zeta_Sa_ system (type II TAc system), a three-component system with a characteristic organization. This system has been located in the multidrug resistance streptococcal plasmid pSM19035 that was chromosomally integrated in the CM05 strain [84]. In this system, the zeta component is the toxin and is inhibited by the antitoxin, the epsilon component, and the operon is regulated by the third component, omega. A 7-bp repeat binding site was identified upstream of the omega site, thus suggesting autoregulation of the operon [24,84]. Additionally, the product of the *omega* gene can participate in partitioning of the plasmid as the ParB protein [85]. Although the function of this system in *S. aureus* is not clear, a role in plasmid stabilization in *Enterococcus* spp. has been demonstrated, as previously discussed [24]. A similar system was studied in *Streptococcus pneumoniae*, the PezAT system, in which the toxin phosphorylates the peptidoglycan precursor UDP-N-acetylglucosamine [86].

#### 1.1.3. *Klebsiella pneumoniae*

*Klebsiella pneumoniae* (order Enterobacteriales) is an important opportunistic pathogen. It is found in the environment, as well as in water or solids and on plant surfaces. Due to increasing levels of antibiotic resistance, this species has become a serious threat to public health throughout the world, causing urinary tract infections, nosocomial pneumonia and intra-abdominal infections. Carbapenem resistant isolates of *K. pneumoniae* were previously associated with numerous infections with few treatment options. The genome of *K. pneumoniae* is extremely plastic [35].

Wei et al. [35] used a bioinformatic approach to analyze the type II TA locus distribution and compared TA diversity in 10 completely-sequenced *K. pneumoniae* genomes. These authors found 212 putative type II TA loci in the *K. pneumoniae* strains. They also showed that some RelBE-like TA groups were distributed differently from the other RelBE systems in *K. pneumoniae*. The RelBE_1_Kp_ and RelBE_2_Kp_ loci were found in the same *K. pneumoniae* isolates, but were distributed differently in plasmids and chromosomes. All members of the RelBE_1_Kp_ group are found in plasmids, while all members of the RelBE_2_Kp_ group are present in chromosomes [35]. The RelBE_Kp_ system has been related to persister cells that are tolerant of antibiotics, such as β-lactams, quinolones and aminoglycosides; more persister cells appeared at high cell densities than at low cell densities [36].

#### 1.1.4. *Acinetobacter baumannii*

*Acinetobacter baumannii* (order Pseudomonadales) is an important pathogen that causes nosocomial infections associated with several types of infections, including pneumonia, meningitis, septicemia and urinary tract infections [87]. Mortality in patients suffering *A. baumannii* infections can be as high as 75% [88]. Several factors have been associated with the pathogenesis of this bacterium: antibiotic resistance, virulence and persistence [89].

Jurenaite et al. [90] used bioinformatics tools to detect the presence of the putative TA loci in *A. baumannii* strains and found at least five functional TA systems. The TA systems differ in their location and abundance and are clearly associated with plasmids. The most commonly-occurring plasmid in TAp systems in *A. baumannii* is plasmid p3ABAYE, of a size of 94 kb, possibly containing five TAp systems. Three of these, RelB/RelE_Ab_ and two versions of HigB/HigA_Ab_, are arranged in opposite directions. The other two are the so-far-unique SplTA (DUF497/COG3514 domain proteins) and CheTA (HTH/GNAT domain proteins) TA systems. In a collection of *A. baumannii* clinical isolates from Lithuanian hospitals (88.6% prevalence among 476 clinical isolates), two of the most abundant TA systems found were the HigB/HigA_Ab_ and SplTA TA systems, which according to the results of the bioinformatic analysis are only plasmid borne. These noncanonical TA systems are the most prevalent in clinical *A. baumannii* isolates belonging to the ECI and ECII lineages, which are spread throughout the world. Interestingly, expression of the HigBA_Ab_ TA system was not revealed by RT-PCR in 46 *A. baumannii* clinical strains [37]. However, Mosqueda et al. [38] located the AbkB/AbkA TA system (so-called SplTA) in the most prevalent plasmid carrying OXA 24/40 ß-lactamase (main mechanism of resistance of carbapenems in *A. baumannii* clinical strains). The AbkB (or SplT) toxin was shown to inhibit translation when overexpressed in *E. coli* with cleavage of lpp mRNA and the transfer of messenger RNA (tmRNA), thus indicating that the AbkB toxin probably functions as an endoribonuclease. The presence of a TA system in these plasmids would explain their stability in the absence of any apparent selection pressure, particularly for small plasmids without the *bla*_OXA24_/*bla*_OXA40_-like gene, such as pAC30a and pAC29a [91].

Interestingly, in a study of the levels of expression of the TAc type II systems in 85 *A. baumannii* clinical isolates, overexpression of the mazEF_Ab_ system was observed in all chromosomal DNA. However, RelBE_Ab_ and HigAB_Ab_ systems showed levels of expression of 88.2% and 4.7%, respectively [37].

#### 1.1.5. *Pseudomonas aeruginosa*

*Pseudomonas aeruginosa* (order Pseudomonadales) is an opportunistic Gram-negative pathogen [92] that causes many chronic infections, including those associated with cystic fibrosis (CF) [93], burn wound infections, bacterial keratitis, urinary infections and peritoneal dialysis catheter infections [92]. Although TA systems are important for infection, few studies have investigated the role of TA systems in pseudomonads, as TA systems have primarily been studied in *E. coli* [93]. This is surprising given that *P. aeruginosa* is the primary model for biofilm formation [94] and that persister cells are prevalent in biofilms, including *P. aeruginosa* biofilms [95].

Three Tap systems (ParAB_Pa_, TOX1/TOX2 and T/AT1-2) were identified but not characterized in 2013, in the pNOR-2000 plasmid encoding *bla*VIM-2, which produces resistance to carbapenems in *P. aeruginosa* clinical strains [40]. The other TA systems described in *P. aeruginosa* clinical strains are located in the chromosome. The presence of TAc systems in the *Pseudomonas* genus was first reported by Williams et al. [40], who showed that the genes for the type II TA systems RelE/RelB_Pa_ and HigB/HigA_Pa_ were present in 42 clinical isolates of *P. aeruginosa*; however, although the authors showed that many of these loci are transcribed, no TA system was verified. The first TA system characterized in pseudomonads was the GraT/GraA TAc system of *P. putida.* This type II TAc is primarily used at low temperatures [41]; the GraT toxin inhibits ribosome assembly at low temperatures by interacting with the DnaK chaperone [45]. The first TA system to be characterized in *P. aeruginosa* was recently reported: the HigB/HigA_Pa_ type II TA system [44]. This TAc system is found in many pathogens; for example, genes for the HigB/HigA-like TA system are found in the Rts1 plasmid of *Proteus vulgaris* and in chromosomes of the pathogens *Vibrio cholera* [96,97], *Streptococcus pneumoniae* [98], *A. baumannii* [90], *Salmonella typhimurium* [99], *Yersinia pestis* [100], *Mycobacterium tuberculosis* [101], *E. coli* CFT073 [102] and *E. coli* O157:H7 [103] and the system is also present in *E. coli* K12 [104]. As noted above, HigBA_Pa_ is also prevalent in *P. aeruginosa* clinical isolates [43]. HigB is an endoribonuclease in *Proteus* spp. [104], *V. cholera* [96], *A. baumannii* [90] and *E. coli* K12 [103]. In *P. aeruginosa*, the HigB toxin is also an endoribonuclease, and the chromosomal HigB/HigA_Pa_ system was found to be a bona fide type II TA system as the antitoxin HigB was shown to mask the toxicity of HigA as a protein and both associated proteins [44]. Critically, the HigB/HigA_Pa_ system affects the virulence factors of *P. aeruginosa*, as activation of the HigB toxin reduces pyocyanin (a toxin produced and secreted by *P. aeruginosa*), the siderophore pyochelin, surface motility (swarming) and biofilm formation [44]. The reduction in pyochelin was also corroborated by a whole-transcriptome study [44]. The HigB/HigA_Pa_ TAc system of *P. aeruginosa* therefore affects the pathogenicity of this strain in a way that has not previously been demonstrated for other TA systems.

Many of the long-term infections produced by *P. aeruginosa*, such as in CF, are due to persister cells [105]. Persister cells are dormant cells [106] that arise without a genetic change in response to stress [107]. Resistant cells grow in the presence of stress factors (such as antibiotics) due to mutations, whereas persisters do not grow and are not affected, due to their metabolic inactivity. Critically, cells become persisters by means of TAc systems [108]; TAc systems cause dormancy by inactivating key metabolic functions, such as protein and ATP production [109]. The link between TAc systems and persister cells was first found through transcriptomics [110,111] and later by deleting single TA systems and showing that this decreases persistence [46,47]. 

#### 1.1.6. *Enterobacter* spp.

We did not find any studies of TA systems in this pathogen.

### 1.2. Other Pathogens 

#### 1.2.1. *Escherichia coli*

*Escherichia coli* (order Enterobacteriales) is an intestinal-dwelling bacterium and an opportunistic pathogen. However, some types can cause illness and diarrhea. Moreover, a strain of *E. coli* called 0157: H7 causes bloody diarrhea and can sometimes cause kidney failure and even death, especially in children and adults with weakened immune systems. The first outbreak of *E. coli* O157: H7, identified in 1982, was associated with eating hamburger meat contaminated with the bacteria [112]. Since then, outbreaks of *E. coli* O157: H7 have been associated with other types of food, such as spinach, lettuce, cabbage and cucumber.

Extended-spectrum beta-lactamase (ESBL)-plasmid encoded ESBL-enzymes, such as CTX-M and TEM, are frequently produced by *E. coli* strains. The following TAp systems are associated with the stabilization of these plasmids (mainly type II): Hok/Sok_Ec_, SrnBC, VagCD_Ec_, PemIK_Ec_, RelBE_Ec_, VapBC_Ec_, CcdAB, MazEF_Ec_, ParAB_Ec_ and PsiAB [51,52].

At least 30 TAc systems are encoded in the *E. coli* K12 genome (chromosome), of which some 12 are well characterized [2]. To date, TA systems of this bacterium are arguably the best characterized and represent current paradigms. Of the five TA types, *E. coli* possesses the type I, II, IV and V systems. Examples of type I TA systems in *E. coli* include the TisB-IstR-1 pair, in which the *tisB* toxin gene is repressed by LexA, so that its expression is de-repressed by DNA damage as part of the SOS response. Under such conditions, enhanced tisB mRNA synthesis out-titrates IstR-1, and tisB RNA is therefore translated and toxin produced, thus decreasing the growth rate and allowing DNA repair mechanisms to act. When conditions return to normal, LexA represses tisB, and the remaining mRNA is rapidly inactivated by IsrR-1 [53]. The TisB toxin acts by destabilizing the inner membrane, probably forming a pore that dissipates the membrane potential, thus inhibiting ATP synthesis [4]. The decrease in ATP concentration then produces an abrupt decrease in transcription and translation rates, and cell replication finally ceases [4]. Another representative example of type I TA systems in *E. coli* is the SymE/SymR pair. As in the case of the *tisB* gene, the *symE* gene encoding the toxin is strongly repressed by LexA, antagonized by the SymR RNA and cleaved by the Lon protease; however, unlike TisB, SymE appears to act as an RNA endonuclease that helps bacteria to get rid of damaged RNA that otherwise could accumulate under SOS activating conditions, rather than acting as a pore forming toxin [54]. Examples of type II TA systems in *E. coli* include the MazE/MazF_Ec_ pair, in which MazF is a toxin with sequence-specific mRNA endoribonuclease activity, and the MazF concentration rises as a consequence of diverse types of stress, such as nutrient starvation, oxidative stress, high temperatures and the presence of bacteriophages [55,56,113]. MazF inhibits translation by cleaving mRNAs at specific sites in a ribosome-independent manner [57,58]. For the type II RelB/RelE_Ec_ pair, the toxin RelE also degrades mRNA at specific sequences; however, in contrast to MazF, it targets RNA when it is bound to the ribosomal A site [59]. Other type II systems in *E. coli* include the YefM/YoeB_Ec_ pair, in which the toxin YoeB (an analogue of RelE) blocks initiation of translation by binding to the 50S ribosomal subunits and then interacts with the A site, promoting the release of the 3′-end portion of the mRNA from the ribosomes [60] and the MqsR/MqsA pair. Interestingly, the *mqsR* gene is the most highly upregulated in *E. coli* persister cells [110] and is also the first TA system associated with biofilms [114]. Furthermore, deletion of *mqsR* provided the first demonstration that a single toxin could be deleted and the number of persister cells reduced, thus linking toxins to persistence [46]. The MqsR tridimensional structure consists of an alpha/beta fold that is homologous with the RelE/YoeB toxins, while MqsA is an elongated dimer that neutralizes MqsR toxicity [115]. In addition to its role as a classic antitoxin, MqsA also works as a global regulator and is the first antitoxin shown to regulate more than its own locus by binding palindromic sequences at other positions on the chromosome [116]. MqsA influences important physiological processes, such as biofilm formation and the global stress response [61]. Interestingly, the MqsR/MqsA TAc system also controls the expression of another toxin/antitoxin system, GhoT/GhoS, as MqsR preferentially cleaves the mRNA of antitoxin GhoS; hence, there is a hierarchy in TA systems as they regulate cell physiology [117]. The GhoT toxin in turn promotes the generation of cell membrane damage that decreases the production of ATP-halting metabolism, thus protecting cells during stress events [62]. One of the possible physiological roles of the MqsR/MqsA pair is to increase bacterial survival in response to the stress produced by bile acid in the gastrointestinal track, as MqsR degrades ygiS mRNA, which encodes a periplasmic protein that promotes the uptake of one of the main components of bile, the deoxycholate salt. Degradation of ygiS mRNA will then decrease the YgiS protein levels and thus decrease the uptake of deoxycholate and increase tolerance to exposure [118]. Another type II TA pair that regulates biofilm formation is Hha-TomB. The Hha toxin decreases biofilm formation by binding the promoters and repressing transcription of the rare codon tRNAs argU, ileX, ileY and proL, as these codons are over-represented in fimbrial genes. Fimbriae production is inhibited by an Hha-mediated decrease in tRNA, leading to decreased biofilm formation. Repression of the transcription of rare codon tRNAs by Hha also promotes cell lysis and biofilm dispersal due to activation of several prophage lytic genes, such as rzpD, yfjZ, appY and alpA, and due to the induction of ClpP/ClpX proteases that activate toxins by degrading several antitoxins [49]. TA pairs are therefore among the most important regulators of *E. coli* physiology, influencing biofilm formation [49,61], stress response [61,113], quorum sensing [119], bacterial persistence [108,110], survival in their natural habitats [118] and perhaps even virulence of pathogenic strains as in the related bacterium *Salmonella* [99]. The scarce, but valuable evidence for a link between TA and virulence in pathogenic *E. coli* strains is summarized in the following paragraph. Although to date there is a lack of studies characterizing TAc functions in *E. coli* pathogenic strains, in 2010 Fozo and coworkers [82] reported that for enterohemorrhagic *E. coli* (EHEC), which is the most common *E. coli* strain producing disease in the U.S., there are at least 26 TA pairs belonging to six distinct TA families. The same authors demonstrated experimentally that two previously uncharacterized putative toxin genes, z3289 and z3290, which occur in a region present in several *E. coli* and Shigella strains, but absent in the genome of the laboratory strain *E. coli* MG1655, are indeed able to halt growth of *E. coli* when overexpressed. Although studies evaluating the role of TA pairs in pathogenic *E. coli* virulence are scarce, in 2012, Norton and Mulvey [50] demonstrated, using a murine infection model, that in the uropathogenic isolate CFT073, the TA YefM-YoeB_Ec_ and YbaJ-Hha pairs are key to virulence, as mutants lacking these systems were outcompeted by the parental strain during bladder colonization. Moreover, in the same study, it was demonstrated that the PasT/PasI TAc pair aids bacterial survival in the kidneys and also increases the formation of persister cells and increases survival during oxidative and nitrosative stress. The study findings suggest that the same or other TA systems found in pathogenic *E. coli* strains may indeed influence virulence and survival within the host during infection and therefore warrant further research.

#### 1.2.2. *Burkholderia* spp.

The *B. cepacia* complex (order Burkholderiales) is a group of 18 closely-related bacterial species that can cause severe lung infections in cystic fibrosis patients (*Burkholderia cenocepacia*) and melioidosis (*Burkholderia pseudomallei*) [120,121]. Despite antibiotic treatment, melioidosis leads to more than 44% mortality in endemic areas, and in the northeast of Thailand, it is the third most frequent cause of death due to infectious diseases. The bacterium is considered to be a potential bioterrorism agent, because it has the ability to infect by air [122].

Agnoli et al. [123] discovered a pC3 plasmid in *Burkholderia cenocepacia* with two putative Tap systems, TAS1 and TAS2, showing homology to the VapBC and HicAB families, respectively. These systems are associated with the stabilization of the pC3 under oxidative, osmotic, high-temperature and chlorhexidine-induced stress. 

Moreover, several TAc modules have been associated with the tolerance of *B. cenocepacia* to multiple antibiotics (development of persister cells), and different type II TA modules have been detected in this bacterium [63].

Eight candidate TAc systems have been located in the genome of *B. pseudomallei*, and five occurred in a genomic island. Of the candidate toxins, BPSL0175 (RelE1_Bp_) and BPSS1060 (RelE2_Bp_) halted growth when expressed in *E. coli*, whereas expression of BPSS0390 (HicA_Bp_) or BPSS1584 (HipA_Bp_) (in an *E. coli* DhipBA background) caused a reduction in the number of culturable bacteria [63]. The HicAB_Bp_ system in this pathogen (homologous to *E. coli*) is associated with bacterial persistence, suggesting that these TAc systems may play a role in human infections [64].

#### 1.2.3. *Streptococcus pneumoniae*

*Streptococcus pneumoniae* (order Lactobacillus) is a pathogen that can cause various infections in humans and severe invasive processes. This almost exclusively human pathogen causes a large number of infections (pneumonia, sinusitis, peritonitis, etc.) and severe invasive processes (meningitis, sepsis, etc.), particularly in the elderly, children and immunocompromised individuals. It is the main causative organism of community-acquired pneumonia [65].

Nieto et al. [124] characterized and carried out functional analysis of the YefM-YoeB_Spn_ TAc system in *Streptococcus pneumoniae*. The mechanism of regulation of this YefM-YoeB_Spn_ TA system was analyzed four years later [66]. Moreover, in *S. pneumoniae*, this TAc system was implicated in pathogenicity, competence, biofilm formation, persistence and an interesting phenomenon called bistability. In this phenomenon, populations of genetically-identical bacteria that grow under stress conditions will separate stochastically into two or more distinct subpopulations [68]. The PezAT TAc system has been characterized in this pathogen (homologous to the epsilon-zeta_Spy_ TA system in *Streptococcus pyogenes* described in plasmid pSM19035 by Behnke and collaborators) [67,86,125,126]. This system has been associated with the development of biofilm formation in *S. pneumoniae* [3]. Fozo et al. subsequently provided the first description of a type I TA system in the chromosome of *Streptococcus pneumoniae* [82]. The RelBE2_Spn_ locus was associated with the survival of *S. pneumoniae* and colonization of humans under unfavorable conditions [127]. Chan et al. applied bioinformatic analysis to 48 pneumococcal strains and described a fourth TA system, Phd-Doc [128]. 

#### 1.2.4. *Mycobacterium tuberculosis*

Tuberculosis (TB) is one of the most important infectious disease killers worldwide. According to the World Health Organization, 9.6 million people were diagnosed with TB, and 1.5 million died from the disease in 2014 [129]. Most TA systems in this genus have been observed in pathogenic strains, suggesting that they are important in the evolution of mycobacteria and in infectious processes [11]. 

More than 80 TAc systems have been described in the *Mycobacterium tuberculosis* chromosome and have been associated with the persistence and establishment of latent infections of this bacterium [130]. Sala et al. identified the TA systems in *M. tuberculosis* H37Rv: most are type II TA systems; three are potentially type IV TA systems; and some have not yet been classified. The following systems have been classified: YefM/YoeB_Mt_ (one system), RelBE_Mt_ (two systems), ParDE_Mt_ (two systems), HigBA_Mt_ (three systems), TAC (toxin-antitoxin-chaperone) in which the chaperones are SecB-like (one system), ten MazEF_Mt_ (10 systems) and VapBC_Mt_ (50 systems) [69]. 

All of the toxins belonging to YefM/YoeB_Mt_ and RelBE_Mt_ in *M. tuberculosis* are upregulated in response to antibiotics, suggesting that they may affect persistence [131,132]. The ParDE2_Ms_ system is being studied by Gupta et al. who have already reported that the parE toxin inhibits bacterial growth in *M. smegmatis*, suggesting its role in dormancy and stress adaptation. According to these authors, the ParDE2 _Ms_ system may be one of the most important elements in tolerance and adaptation to stress [70]. Three of the systems in *M. tuberculosis* are included in the HigBA_Mt_ family, two of which have not yet been studied in detail in mycobacteria, although researchers suspect their involvement in dormancy [133,134]. The TAC_Mt_ system is regulated by the interaction between chaperone and antitoxin, thus preventing degradation of the chaperone. This system has been demonstrated to be highly conserved in the *M. tuberculosis* complex, suggesting an important function, in accordance with the increased activity in response to DNA damage, heat shock, nutrient starvation, hypoxia, drug-persistence and host phagocytes [71,73]. The second family system with most members in *M. tuberculosis* is MazEF_Mt_. Toxins of this family have been shown to have different targets, suggesting multiple responses: e.g., MazF6 toxins have the capacity to act on ribosomal RNA by cleaving 23S rRNA of dissociated ribosomes, which leads to general inhibition of protein synthesis [73]; the MazF4 toxin interacts with DNA topoisomerase I, thus inhibiting growth [74]. It has also been demonstrated that this system may interact with other bacterial systems, e.g., the MazF9 toxin can be neutralized by antitoxins from the VapBC system, which may be explained by the low percentage of conservation between antitoxins of this system [135]. Most TA systems in *M. tuberculosis* belong to the VapBC family. This group is characterized by a PIN domain, which is present in the toxin component; the PIN domain is homologous to the type IV pili N-terminal domain [136]. These systems produce a response to stress conditions, such as hypoxia (systems 15, 7 and 25) and the presence of macrophage enzymes (systems 11, 3 and 47), supporting the idea that these systems play a role in persistence [69]. Lee et al. demonstrated that one of these enzymes, VapC30, uses magnesium as a cofactor to its ribonuclease activity in inhibiting cellular growth [136]. Some authors also suggest its involvement in the first steps of latent infection [137]. 

## 2. Discussion

In this article, we reviewed the information available to date about TA systems in clinical pathogens. TA systems are involved in some types of bacterial behavior, such as plasmid maintenance, biofilm formation, phase variation, virulence regulation, genetic competence, persister cells [75], regulation of the SOS response and bacterial defense against bacteriophages (abortive infection). 

In relation to plasmid maintenance, most of the pathogens in the ESKAPE group contain plasmids with resistance genes carrying TA systems. In vancomycin-resistant strains of *E. faecium* and *E. faecalis*, the pRUM and pVEF3 plasmids have Axe/Txe_Ef_ and omega/epsilon/zeta_Ef_ type II TA systems, respectively [22,23,80]. On the other hand, the AbkB/AbkA type II TA system in plasmids carrying OXA 24/40 ß-lactamase in *A. baumannii* clinical strains [38], as well as the Hok/Sok_Ec_, SrnBC, VagCD_Ec_, PemIK_Ec_, RelBE_Ec_, VapBC_Ec_, CcdAB, MazEF_Ec_, ParAB_Ec_ and PsiAB systems, which were analyzed in ESBL-plasmids carrying CTX-M/TEM enzymes and isolated from *E. coli* strains, and finally, the ParAB_Pa_, TOX1/TOX2, T/AT1-2 systems have all been found in the pNOR-2000 plasmid encoding *bla*VIM-2 in clinical strains of *P. aeruginosa* [40]. Moreover, type II TA systems have been described in plasmids from environmental isolates, such as hyperthermophilic environments around hydrothermal vents located in the Atlantic, Pacific and Indian Oceans [138]. The plasmids can be ascribed to two subfamilies: pTN2-like and pEXT9a-like. Both plasmids encode TA systems of two different families: VapBC and RelBE. Moreover, other type II TA systems, such as VagCD, have been identified in IncF antibiotic-resistant and virulent plasmid pRSB225, isolated from an unknown bacterium released to the environment via the purified wastewater from a municipal sewage treatment plant [139]. Interestingly, modules of plasmid pRSB225 are associated with segments of different virulence plasmids harbored by entero-aggregative-hemorrhagic *E. coli* (EAHEC). 

The relationship between biofilm formation and TA systems has been widely studied, particularly in *E. coli* [14,17,19,77] and *P. aeruginosa* [44]. In *E. coli,* several type II TA systems have been associated with biofilm formation, MqsR/MqsA, Hha/TomB, MazEF_Ec_, RelBE_Ec_, YefM/YoeB_Ec_, DinJ/JafQ and GhoTS_Ec_, while only the HigBA_Pa_ TA system has been associated with clinical strains of *P. aeruginosa* and biofilm formation. Nevertheless, further experimental studies may discover yet unknown relationships between TA systems in *P. aeruginosa* and biofilm formation persistence and virulence. 

Other functions of TA systems include phase variation and genetic competence, although so far, only the YefM-YoeB_Sp_ system in *S. pneumoniae* has been associated with these functions [66,140]. In 2012, Bukowski M. et al. analyzed the participation of the PemIK_Sa_ system located in the pCH91 plasmid of *S. aureus* in the global regulation of staphylococcal virulence by altering the translation of large gene pools [26]. 

Consistent with the role of TA systems and persistence, TA systems have been associated with disease. For example, inactivation of three type II MazEF_Mt_ TA systems in *Mycobacterium tuberculosis* reduces its pathogenicity in macrophages and in the spleen and lungs of guinea pigs [141]. TA systems have also been found to affect the persistence of *Salmonella typhimurium* in macrophages in a mouse model of typhoid fever [142]. Furthermore, inactivation of Vap-type TA type II systems (VapBC-1 and VapXD TA loci) reduced virulence for non-typeable *Haemophilus influenzae* (NTHi) in a chinchilla model of otitis media [48], and as already discussed, inactivation of YoeB/YefM, Hha/TomB and PasT/PasI type II TA systems proved important for uropathogenic *E. coli* infections of the bladder and kidney in murine models [50]. In *S. aureus*, the MazEF_Sa_ TA system has been associated with persistence [143] and virulence [144], while GhoTS_Ec_ and RelBE_Ec_ in *E. coli* [6,14,145] and RelEB_Pa_ and HigBA_Pa_ in *P. aeruginosa* [44,59] have been associated with the same. The TisB/IstR and SymER TA type I systems have been associated with the regulation of the SOS response in *E. coli* [4,53,54]. Finally, type I, II, III and IV TA systems have been associated with the phage abortive infection system. Among these, we can highlight the following: Hok/Sok_Ec_ (type I TA system), which was the first TA system to be related to phage inhibition [146]; MazF/MazE_Ec_ (type II TA system) [56]; ToxIN (type III TA system), found in *E. coli* strains [147,148]; AbiQ (type III TA system), studied in non-clinical strains, such as *Lactococcus lactis* and *Lactobacillus pentosus* KCA1 isolates [149,150]; and AbiEii (type IV TA system) in *E. coli* samples [151].

These functions or features have been associated with the phylogeny of the species in *E. coli* clinical strains, and the type II TA systems are also involved [152]. There is a potential link between chromosomal type II and *E. coli* phylogeny, with a small number found in group B2 (the main phylogenetic groups of *E. coli* clinical strains are A, B1, B2 and D).

In conclusion, further studies of these systems must be carried out in order to identify other TA pairs and to better define the role of these systems in bacterial virulence. For instance, the genetic diversity of *E. coli* is remarkably high, with a core genome (common to all strains) of approximately 2200 genes, while the accessory genome is much larger, comprising around 13,000 genes, many of which may be involved in bacterial virulence [153]. We hypothesize that some of these encode TA pairs that may be very important during infections. Given the importance of TA systems for the survival of pathogenic bacteria and infections, we propose that these systems could be exploited as novel targets for developing new anti-infectious treatments. This is an urgent need considering the accelerated rate of acquisition of multidrug resistance by intra-nosocomial strains, especially as their inactivation may lead to simultaneous decreases in infectivity, biofilm formation, resistance to stress and antibiotic persistence. In addition, as TA genes are absent in mammalian hosts, anti-TA drugs may be highly specific. Nevertheless, one possible drawback to the effectivity of these types of therapy is the high abundance of these systems in the genome, so that it would be necessary to develop broad spectrum anti-TA, or to simultaneously target several systems, or target those potentially controlling the expression of other TA systems. Another drawback is that it is not sufficient to simply inactivate antitoxins to activate toxins, as this would lead to an increase in persister cells because activation of a toxic protein produces persisters [154]; hence, the toxins would have to be inactivated by the new pharmaceuticals. Some side effects of these treatments may be generated due to inactivation of TA systems of bacterial species belonging to the normal microbiota. Encouraging further basic research on TA systems in different bacteria may provide valuable information for future therapeutic alternatives.

## Figures and Tables

**Table 1:** Role of different types of TA (Toxin-antitoxin) systems in clinical pathogens. ESKAPE group (*Enterococcus faecium*, *Staphylococcus aureus*, *Klebsiella pneumoniae*, *Acinetobacter baumannii*, *Pseudomonas aeruginosa* and *Enterobacter* spp.) and other pathogens of clinical interest.

Bacterium	TA System	Type	Localization	Function	Other Pathogens	References
ESKAPE Group						
***Enterococcus* spp.**	Par locus	I	Plasmid pAD	Regulation, persistence and plasmid maintenance	*Lactobacillus casei* *S. aureus*	[20,21]
	Axe/Txe	II	Plasmid pRUM	Plasmid maintenance and vancomycin resistance (VanA enzyme)	*S. aureus* *E. coli*	[22]
	Omega/Epsilon/Zeta	II	Plasmid pVEF3	Plasmid maintenance vancomycin resistance (VanA enzyme)	*Bacillus subtilis* *E. coli* *S. aureus*	[23,24]
	HigBA	II	Chromosome	Expression of virulence factors	*Enterococcus* spp. *Proteus vulgaris* *Vibrio cholerae* *E. coli* *S. pneumoniae* *A. baumannii* *P. aeruginosa* *Salmonella typhimurium* *Yersinia pestis* *M. tuberculosis*	[25]
	MazEF	II	Chromosome	Expression of virulence factors	*S. aureus* *E. coli* *S. typhimurium* *P. aeruginosa* *M. Tuberculosis*	[25]
***S.aureus***	PemIK	II	Plasmid cCHP91	Plasmid maintenance and global regulation of virulence	-	[26]
	SprG1/Spr1	II	Phage	Lytic activity (human erythrocytes)	-	[27,28]
	SprA1 (PepA1/AS)	I	Pathogenicityisland/Chromosome	Persistence and pathogenicity	-	[29]
	MazEF	II	Chromosome	Regulation of β-lactamase sensitivity	*Enterococcus* spp. *E. coli* *P. aeruginosa* *S. pneumoniae* *M. tuberculosis*	[30,31,32]
	YefM/YoeB	II	Chromosome	Cell arrest	-	[33,34]
	Omega/Epsilon/Zeta		Chromosome	-	*Bacillus subtilis* *E. coli*	[29]
***K. pneumoniae***	RelBE_1	II	Plasmid	Persistence of cells against antibiotics and plasmid maintenance	A. *baumannii* *P. aeruginosa* *E. coli* *B. pseudomallei* *S. pneumoniae*	[35,36]
	RelBE_2	II	Chromosome		*M. tuberculosis*	
***A. baumannii***	RelBE, HigBA, SplTA and CheTA	II	Plasmid p3ABAYE	Plasmid maintenance	*Enterococcus* spp. *Proteus vulgaris* *Vibrio cholerae* *E. coli* *S. pneumoniae* *P. aeruginosa* *Salmonella typhimurium* *Yersinia pestis* *M. tuberculosis*	[37]
	AbKAB (SplTA)	II	Plasmid	Plasmid maintenance and carbapenem resistance (OXA 24 ß-lactamase)	*E. coli*	[38]
	GraTA	II	Plasmid	-	*P. aeruginosa*	[39]
	MazEF	II	Chromosome	-	*Enterococcus* spp. *S. aureus* *P. aeruginosa* *E. coli* *S. typhimurium* *M. tuberculosis*	[37,39]
***P.aeruginosa***	ParAB, TOX1/TOX2, T/AT1-2	II	Plasmid pNOR-2000	Plasmid maintenance and carbapenem resistance(VIM metallo-ß-lactamase)	*Enterococcus* spp. *Lactobacillus casei* *S. aureus*	[40]
	RelBE	II	Chromosome	-	*K. pneumoniae* *A. baumannii* *E. coli* *B. pseudomallei* *S. pneumoniae* *M. tuberculosis*	[41,42,43]
	HigBA	II	Chromosome/ Plasmid Rts1	Reduction of pyochelin, swarming and biofilm formation	*Enterococcus* spp. *P. vulgaris* *V. cholerae* *S. pneumoniae* *A. baumannii* *E. coli* *S. typhimurium* *Yersinia pestis* *M. tuberculosis* *E. coli*	[42,44]
	GraTA	II	Chromosome	Persistence	*A. baumannii*	[45]
	MazEF	II	Chromosome	Persistence	*Enterococcus* spp. *S. aureus* *E. coli* *S. typhimurium* *M. tuberculosis*	[46,47]
	Vap-type systems	II	-	Regulation of virulence	*Haemophilus influenzae*	[48]
	YefM/YoeB	II	Chromosome	Regulation of virulence	*E. coli* *S. aureus* *S. pneumoniae* *M. tuberculosis*	[49]
	Hha/TomB	II	Chromosome	Regulation of virulence	*E. coli*	[49]
	PasTI	II	Chromosome	Regulation of virulence	*E. coli*	[50]
***Enterobacter* spp.**	-	-	-	-		-
**Other pathogens**						
***E.coli***	PemIK, VagCD, CcdAB, Hok/Sok, ParAB and PsiAB	II	Plasmid pEC302104	Plasmid maintenance and ß-lactam resistance (ESBL ß-lactamase)	*Enterococcus* spp. *S. aureus*	[51,52]
	TisB/IstR	I	Chromosome	Regulation of SOS response	-	[4,53]
	SymER	I	Chromosome	Regulation of SOS response	-	[54]
	MazEF	II	Chromosome	Persistence, biofilm formation	*Enterococcus* spp. *S. aureus* *S. typhimurium* *P. aeruginosa* *M. tuberculosis*	[55,56,57,58]
	RelBE	II	Chromosome	Persistence, biofilm formation	*K. pneumoniae* *A. baumannii* *P. aeruginosa* *B. pseudomallei* *S. pneumoniae* *M. tuberculosis*	[59]
	YefM/YoeB	II	Chromosome	Persistence, biofilm formation	*S. aureus,* *P. aeruginosa* *S. pneumoniae*	[60]
	MqsRA	II	Chromosome	Influence on biofilm formation and global stress response. Control of GhoTS System. Increased tolerance to bile acid.	-	[49,50,51,52,53,54,55,56,57,58,59,60,61]
	GhoTS	V	Chromosome	Persistence, biofilm formation	-	[49,62]
	Hha/TomB	II	Chromosome	Persistence, decreases biofilm formation by inhibiting fimbriae production.	*P. aeruginosa*	[49,61]
	PasTI	II	-	Persistence	*P. aeruginosa*	[50]
***Burkholderia* spp.**	TAS1/TAS2	II	Plasmid pC3	Plasmid maintenance and tolerance to antibiotics	-	[63,64]
	RelBE	II	Chromosome	Persistence	*K. pneumoniae* *A. baumannii* *P. aeruginosa* *E. coli* *S. pneumoniae* *M. tuberculosis*	[63]
	HicAB	II	Chromosome	Persistence	*E. coli*	[64,65]
***Streptococcus* spp.**	YefM/YoeB	II	-	Implicated in pathogenicity, phase variation, genetic competence, biofilm formation and bistability	*E. coli* *S. aureus* *P. aeruginosa* *M. tuberculosis*	[66,67]
	PezAT	II	-	Persistence and biofilm formation	-	
	RelBE	II	Chromosome	Associated with survival and human colonization	*K. pneumoniae* *A. baumannii* *P. aeruginosa* *E. coli* *B. pseudomallei* *M. tuberculosis*	[66]
	Phd-Doc	-	-		-	[68]
***M.tuberculosis***	YefM/YoeB	II	Chromosome	Persistence	*E. coli* *S. aureus* *P. aeruginosa* *S. pneumoniae*	[69,70,71]
	RelBE	II	Chromosome	Persistence	*K. pneumoniae* *A. baumannii* *P. aeruginosa* *E. coli* *B. pseudomallei* *S. pneumoniae*	[70,71]
	ParDE	II	Chromosome	Persistence	-	[72]
	HigBA	II	Chromosome	Persistence	*Enterococcus* spp. *P. vulgaris* *V. cholerae* *A. baumannii* *E. coli* *S. pneumoniae* *Salmonella typhimurium* *Yersinia pestis*	[73,74]
	TAC		Chromosome	-	-	[69]
	MazEF	II	Chromosome	Persistence and cell arrest	*Enterococcus* spp. *S. aureus* *E. coli* *S. typhimurium* *P. aeruginosa*	[75]
	VapBC	II	Chromosome	Persistence	*P. aeruginosa* *H. influenzae*	[17,76,77]
